# Velocity-Adaptive V2I Fair-Access Scheme Based on IEEE 802.11 DCF for Platooning Vehicles

**DOI:** 10.3390/s18124198

**Published:** 2018-11-30

**Authors:** Qiong Wu, Siyang Xia, Pingyi Fan, Qiang Fan, Zhengquan Li

**Affiliations:** 1 Jiangsu Provincial Engineering Laboratory of Pattern Recognition and Computational Intelligence, Jiangnan University, Wuxi 214122, China; qiongwu@jiangnan.edu.cn (Q.W.); siyangxia@stu.jiangnan.edu.cn (S.X.); 2 National Mobile Communications Research Laboratory, Southeast University, Nanjing 210096, China; 3 Department of Electronic Engineering, Tsinghua University, Beijing 100084, China; 4 Advanced Networking Lab., Department of Electrical and Computer Engineering, New Jersey Institute of Technology, Newark, NJ 07102, USA; qf4@njit.edu

**Keywords:** autonomous driving, platoon, IEEE 802.11 DCF, fair-access

## Abstract

Platooning strategy is an important component of autonomous driving technology. Autonomous vehicles in platoons are often equipped with a variety of on-board sensors to detect the surrounding environment. The abundant data collected by autonomous vehicles in platoons can be transmitted to the infrastructure through vehicle-to-infrastructure (V2I) communications using the IEEE 802.11 distributed coordination function (DCF) mechanism and then uploaded to the cloud platform through the Internet. The cloud platform extracts useful information and then sends it back to the autonomous vehicles respectively. In this way, autonomous vehicles in platoons can detect emergency conditions and make a decision in time. The characteristics of platoons would cause a fair-access problem in the V2I communications, i.e., vehicles in the platoons moving on different lanes with different velocities would have different resident time within the infrastructure’s coverage and thus successfully send different amounts of data to the infrastructure. In this case, the vehicles with different velocities will receive different amounts of useful information from the cloud. As a result, vehicles with a higher velocity are more likely to suffer from a traffic accident as compared to the vehicles with a lower velocity. Hence, this paper considers the fair-access problem and proposes a fair-access scheme to ensure that vehicles with different velocities successfully transmit the same amount of data by adaptively adjusting the minimum contention window of each vehicle according to its velocity. Moreover, the normalized throughput of the proposed scheme is derived. The validity of the fair-access scheme is demonstrated by simulation.

## 1. Introduction

Autonomous driving technology has become one of the hottest research topics in recent years. Automakers such as Volkswagen and General Motors expect that autonomous vehicles will be available on the market in 2020 and 25 percent of vehicles on the road will become autonomous vehicles by 2035 [1]. Compared with traditional vehicles, autonomous vehicles have the following advantages: thousands of lives can be saved by improving the road safety; fuel consumption can be reduced by alleviating traffic jams; users’ demand can be satisfied by liberating them from a long time’s drive, enabling them to do something freely [2].

Platooning is an important autonomous driving management strategy [3]. With the platooning strategy, autonomous vehicles form a group on a common lane, which we refer to as a platoon being composed of a leader vehicle and several member vehicles. The leader vehicle leads a platoon and controls the kinematics parameters of the platoon, e.g., velocity and acceleration. Member vehicles follow the leader vehicle one after another [4]. To keep a platoon formation, autonomous vehicles in a platoon exchange their kinematics information through vehicle-to-vehicle (V2V) communications. In this case, vehicles in a platoon keep moving at a constant speed and have a small constant inter-vehicle spacing [5]. In addition, to perceive the surrounding environment and react promptly according to emergency conditions, autonomous vehicles in a platoon are often equipped with a variety of on-board sensors, e.g., various cameras and LiDARs, to collect the ambient information about road conditions, pedestrians, and other vehicles. Different from the traditional sensors, the outfitted cameras capture high quality image containing abundant information with the rate of 100–700 Mb/s and LiDARs generate high resolution maps with the rate of 10–100 Mb/s [6]. The abundant information is usually redundant and needs to be stored, computed, and analyzed to extract the useful information [7]. However, the storage, computation and analysis abilities of an autonomous vehicle are restricted. To solve this problem, one autonomous vehicle can deliver these large amounts of data to the infrastructure through vehicle-to-infrastructure (V2I) communications, and then the infrastructure uploads the large amounts of data to the cloud platform via the Internet. Thus, the cloud platform uses its strong computation and storage capacity [8] to abstract useful information and then sends it back to the autonomous vehicles. In this way, autonomous vehicles can sense emergency conditions and make a timely decision. Cooperatively considering V2V and V2I communications can reduce the amount of data uploaded and downloaded through multi-hop communication between vehicles [9,10]. In this paper, we investigate the one-hop V2I communication as with many related works which are devoted to solving the problems in one-hop communication scenario [11,12,13].

The autonomous vehicles in a platoon usually employ the IEEE 802.11 distributed coordination function (DCF) mechanism [14] to access the infrastructure with the same access parameters including the minimum contention window size and maximum back-off stage [15]. On the other hand, platoons have some unique characteristics, e.g., the velocity of vehicles moving on a common lane is constant; the velocities of vehicles moving on different lanes may be different; the inter-vehicle spacing is also constant and related to the velocity. These characteristics would affect the effectiveness of the V2I communications when the IEEE 802.11 DCF mechanism is adopted. Owing to the above two factors, the fair-access problem occurs in the V2I communications. Specifically, vehicles moving on a common lane with a high velocity would take a short time to traverse the coverage of one roadside infrastructure, while vehicles moving on another lane with a low velocity would take a long time. Thus, vehicles moving on different lanes would have different resident time within the coverage of the roadside infrastructure and thus send different amounts of data successfully to it. Afterwards, the roadside infrastructure uploads the data to the cloud platform that stores, computes, and analyzes the data to abstract the useful information and then the cloud platform sends it back to the corresponding vehicles. Thus, the vehicles with different velocities will receive different amounts of useful information. As a result, vehicles with a higher velocity are more likely to suffer from a traffic accident because they receive less useful information about the traffic condition.

Our previous work has considered the fair-access problem in VANETs [16]. In [16], we assumed that the vehicles moving on different lanes with the same velocity are grouped in batches and the vehicles in each batch arrive at the coverage of an infrastructure at the same time. However, it did not consider the characteristics of platoons mentioned above. Several works have focused on the study of platoons, but these works did not consider the fair-access problem in platoons [17,18,19,20,21,22,23,24]. To the best of our knowledge, there is no research proposing a scheme to solve the fair-access problem for platoons. This is the motivation of our work.

In this paper, we concentrate on addressing the aforementioned fair-access problem, under the condition of multi-platoons on different lanes. To solve the problem, we propose a velocity-adaptive V2I fair-access scheme based on IEEE 802.11 DCF for platooning vehicles to guarantee the fairness among vehicles with different velocities on different lanes. We first define a fair-access index, which means that the vehicles fairly access the roadside infrastructure as long as they have the same fair-access index. Next, we discuss the relationship between the average number of vehicles and the vehicle velocity on the same lane. Then, we derive the relationship among the transmission probability, the vehicle velocity and the minimum contention window for transmission. Finally, we derive the relationship among the fair-access index, the vehicle velocity, and the minimum contention window for transmission. In this way, the fair-access indexes of vehicles can be guaranteed to be equal by dynamically adjusting the minimum contention window of each vehicle according to its velocity. In addition, we derive the normalized throughput of the proposed scheme. The validity of our scheme is proven by simulation. The main contributions of our paper can be summarized as follows:

1. This paper focuses on the V2I communication issues in autonomous driving platoons and we are committed to addressing the V2I fair-access problem caused by different platoon velocities.
2. We build an analytical model based on the scenario of multi-platoon on different lanes with various parameters, e.g., vehicle velocity, intra-platoon spacing, inter-platoon spacing and platoon arrival rate, to guarantee the fairness among vehicles with different velocities on different lanes.
3. We propose a velocity-adaptive V2I fair-access scheme based on IEEE 802.11 DCF for platooning vehicles. By dynamically adjusting the minimum contention window to keep a fairness index unchanged, our proposed scheme can ensure that all vehicles with different velocities in the coverage of the roadside infrastructure access the roadside infrastructure fairly.
4. We verify the effectiveness of our scheme through simulation. Moreover, using this analytical model, we analyze the system performance in this scenario.

The rest of the paper is organized as follows. Section 2 reviews the related work of platooning and approaches to fair-access problems in the past few years. Section 3 depicts the system model. Section 4 details the analytical model of velocity-adaptive V2I fair-access scheme and analyzes the performance of network throughput. Section 5 presents the simulation results. The conclusions are given in Section 6.

## 2. Related Work

In this section, we review the recent works related to platoon communications and various fair problems.

The platoon communications have drawn much attention recently. Various methods have been proposed to improve the performance of the platoon communications [17,18,19,20,21,22,23,24]. In [17], Ucar et al. considered the directionality and impermeability of light and proposed a security protocol based on 802.11p standard for platoon communications. The proposed protocol can compensate the characteristics of visible light communication that is sensitive to the environment so that the stability of platoons could be improved. In [18], Gao et al. considered a consistent communication delay for different kind of vehicles in a platoon, e.g., truck, sedan, and coach, and presented an H-infinity control strategy for the platoon formation. This method satisfied the requirement of linear stability and robustness to resist the uncertainties of vehicle dynamics in the platoon. In [19], Guo and Wen considered the capacity limitation of wireless communication channels in VANETs and established a framework of access scheduling based on the scheduling sequences to address the network access conflict and reduce the zero steady-state spacing error. Simultaneously, this article modeled the random packet loss as an independent Bernoulli process to analyze the performance of the platoon. In [20], Xu et al. examined the impact of packet loss on vehicle safety in platoon communications with the condition of block erase channels. Considering the structural characteristics of different information obtained from radar distance sensors and wireless communication channels, the authors derived the relationship between the communication performance and control parameters to guarantee the cooperative communication and safety in platoons. In [21], Peng et al. presented an LTE-based sub-channel allocation scheme and power control mechanism for intra-platoon and inter-platoon communications under a multi-platoon scenario. This work considered multicast and device-to-device (D2D) communications and achieved extremely low delays via reasonable cellular resource allocation. In [22], Kazemi et al. proposed a neural network-based entry detection and trajectory prediction scheme for the sudden cut-in behavior in the cooperative adaptive cruise control (CACC) system, where vehicle-to-vehicle (V2V) communication is used to improve the response accuracy of the platoon system. In [23], Campolo et al. investigated the potential of data dissemination in the platoon when using long-term evolution (LTE) device-to-device (D2D) communication mode. It can be seen that when this communication mode is adopted, the cooperative awareness messages (CAMs) exchanged in a platoon have a great spatial reuse of LTE resources in both the same platoon and different platoons with ultra-low latency. In [24], Gao et al. proposed a distributed adaptive sliding control strategy to address the platoon formation problem arising from the changing network topology which causes the unstable communication channel. Moreover, an algorithm was proposed based on the linear matrix inequality to enable the vehicles to drive cooperatively and form a platoon dynamically. All the works mentioned above emphasized the implementation of platooning in autonomous driving. However, none of them considered the fair problem for the communications of platoons.

In recent years, several works have designed schemes to solve various fair problems in wireless network. In [25], Rastegar et al. established a fair allocation scheme regarding of flow table spaces to reduce the delay of users in software defined radio (SDR) access networks when user information is obtained from the controller. In [26], Yang et al. used logarithmic utility functions to ensure the user fairness in light modulation of the multi-user visible light communications (VLC). Then, they proposed a low-complexity optimal power control algorithm to maximize the total system throughput. In [27], Cha et al. considered frame error rates and proposed a novel media access control (MAC) protocol to guarantee the fair channel access in uplinks to support multi-packet reception. In [28], Iosifidis et al. studied the fair-access scheme to share frequency channels in the network where LTE technologies coexist with Wi-Fi technologies. However, these works did not consider the fair-access problem, in which the amount of data successfully delivered from vehicles to the cloud platform is impacted by the velocity of vehicles. In [29], Xiong et al. designed a fairness-adjustable time-domain power allocation approach towards 5G high mobility.

Our previous work proposed a fair channel access scheme for the V2I communication based on the IEEE 802.11 DCF in VANETs [16]. In [16], it assumed that vehicles on different lanes with the same velocity are grouped in batches and the vehicles in each batch arrive at the coverage of one roadside infrastructure at the same time. However, in the platoon scenario, vehicles in platoons move on different lanes with different velocities and the vehicles in a platoon arrive at the coverage of the roadside infrastructure one by one. Therefore, the assumption in our previous work is not suitable for the platoon scenario. As far as we know, no one has jointly considered the characteristics of platoons and the fair-access problem in the platoon scenario. Thus, we design a fair-access scheme to guarantee that the amount of data successfully delivered by each vehicle in all platoons should be equal by dynamically adjusting the minimum competition window according to vehicles’ velocities under non-saturated conditions.

## 3. System Model

**Figure 1:**
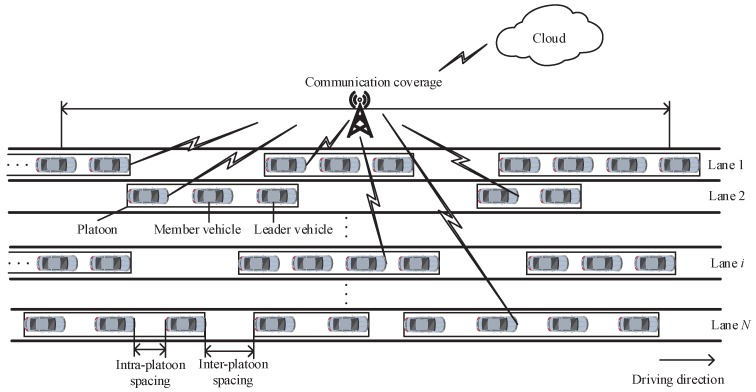
System model.

Consider a system model as shown in Figure 1. Multiple platoons move on a highway with multiple straight lanes covered by one roadside infrastructure. Each platoon consists of a leader vehicle and some member vehicles. A leader vehicle controls the velocity of a platoon. The member vehicles follow the leader vehicle one after another on the same lane in a queue with the same velocity and keep a constant intra-platoon spacing, i.e., the distance between two consecutive vehicles in a same platoon. Each vehicle does not change its moving direction. The platoons on the same lane move with a specified velocity and arrive at the coverage of the roadside infrastructure according to a Poisson process with an arrival rate. The platoon arrival rate should be smaller than a limit to guarantee the average inter-platoon spacing, i.e., the average distance between the last vehicle of the preceding platoon and the leader vehicle of the following platoon on the same lane, is larger than the intra-platoon spacing to avoid collision [30]. The specified velocity and platoon arrival rate of the platoons are different for different lanes.

It assumes that a communication transceiver is installed at the headstock of each vehicle. Each vehicle transmits packets to the infrastructure once the headstock of the vehicle arrives at the coverage of the roadside infrastructure. This paper considers a non-saturated condition, i.e., each vehicle does not always have packets to transmit [31]. A vehicle usually transmits packets in the Control Channel (CCH) and the Service Channel (SCH). However, the control and safety messages transmitted in the CCH are very short [32]. Thus, the amount of data transmitted in CCH is not large. In this case, the difference of the amount of data successfully transmitted in the CCH by the vehicles with different velocities is not obvious. On the other hand, the messages transmitted in the SCH are often service information and the amount of data transmitted in the SCH are usually very large, e.g., as mentioned in Section 1, an autonomous vehicle equipped with various sensors generates large amounts of data with the rate of about 100–700 Mb/s. As a result, when considering the fair-access problem caused by different velocities, the control messages transmitted in the CCH are not so important, compared to the large amount of data transmitted in the SCH. Therefore, we only consider the SCH for transmitting the abundant data in this paper. The IEEE 802.11 DCF mechanism is adopted in the SCH to transmit packets. That is, when a vehicle has a packet to transmit, the packet will be transmitted if the channel keeps idle within a DIFS (distributed inter-frame space) duration. Otherwise, if the channel is busy in a slot of the DIFS duration, a back-off procedure will be initialized. In this case, a back-off counter is started up with an integer randomly selected from [0, CW−1]. Please note that CW = $CW_{min}$, CW is the contention window and $CW_{min}$ is the minimum one. Then the back-off counter will be decremented by 1 after the channel is detected as idle in a slot. When the back-off counter is decreased to be 0, the packet will be transmitted. Afterwards, if the vehicle does not receive an ACK (acknowledgement) packet after the SIFS (short inter-frame space) duration, the back-off stage, i.e., the number of retransmission times, is incremented by 1 while another new back-off procedure with a doubled CW is initialized to retransmit the packet. When the back-off stage reaches the maximum back-off stage *m*, the contention window CW will become the maximum contention window $CW_{max}$, which will be kept at each retransmission. If the packet is transmitted successfully, i.e., the vehicle receives an ACK packet after the SIFS duration, the value of CW is reset to $CW_{min}$ and the back-off stage is reset to 0, then a new back-off procedure is initialized. If the vehicle has no packet to transmit and the back-off counter is decremented to zero, it will keep this state until another packet arrives.

When each vehicle adopts the IEEE 802.11 DCF mechanism to transmit packets, the access parameters including the minimum contention window $CW_{min}$ and the maximum back-off stage *m* are the same for each vehicle. In this case, the vehicles moving on a lane with a higher velocity would transmit a smaller amount of data successfully than the vehicles on another lane with a slower velocity because they stay less time within the coverage of the roadside infrastructure, thus incurring a fair-access problem. In this paper, we propose a fair-access scheme based on the IEEE 802.11 DCF mechanism. The fair-access scheme adaptively adjusts the minimum contention window size according to each vehicle’s velocity to ensure that vehicles with different velocities successfully send the same amount of data in the V2I communications. In Section 4, we will derive the velocity-adaptive minimum contention window size in detail.

## 4. Analytical Model of Velocity-Adaptive V2I Fair-Access Scheme

In this section, we consider the system model described in Section 3 and propose a velocity-adaptive V2I fair-access scheme to ensure that vehicles with different velocities successfully send the same amount of data in the V2I communications. Specifically, the minimum contention window size of each vehicle is adjusted adaptively according to its velocity to guarantee that the amount of successfully delivered data is equal for each vehicle. We first derive a fair-access index by using the average number of vehicles on each lane and the transmission probability of a vehicle to evaluate the fairness of the proposed scheme. To obtain the relationship among the fair-access index, minimum contention size and a vehicle’s velocity, we further derive the relationship between the average number of vehicles and a vehicle’s velocity on a lane, and the relationship among the transmission probability, vehicle velocity and minimum contention window on a lane. After that, according to the derived relationships, we adjust the minimum contention window size of each vehicle adaptively according to its velocity to achieve fairness. Finally, we also derive the normalized throughput of the proposed scheme. The parameters used in the analytical model are listed in Table 1.

### 4.1. Fair-Access Index

In this sub-section, we define a fair-access index to evaluate the fairness of the proposed scheme. First, we assume that vehicles on the same lane have the same parameters. For the sake of fair access, the total amount of data successfully transmitted within the resident time of any vehicle on each lane should be equal. Therefore, we have

(1) $$R_{s}^{i}T_{i}=C,$$

where $R_{s}^{i}$ is the successful transmission rate of a vehicle on lane *i* (*i* = 1, 2, ⋯, *N*), $T_{i}$ is the residence time of a vehicle on lane *i*, and *C* is a constant.

According to the transmission rate of a vehicle calculated in [16], the successful transmission rate of a vehicle on lane *i* can be calculated as

(2) $$R_{s}^{i}=H\times \frac{R_{bit}}{N_{bit}}\times \frac{p_{s}^{i}}{\sum_{j=1}^{N_{v}}\tau_{j}}.$$

Substituting Equation (2) into (1), we have

(3) $$H\times \frac{R_{bit}}{N_{bit}}\times \frac{p_{s}^{i}}{\sum_{j=1}^{N_{v}}\tau_{j}}\times T_{i}\approx C.$$

In Equation (3), *C*, *H*, $R_{bit}$, $N_{bit}$ and $\sum_{j=1}^{N_{v}}\tau_{j}$ are all constant. Therefore, we define the fair-access index as

(4) $$K_{index}\approx p_{s}^{i}T_{i},T_{i}=\frac{D}{v_{i}},$$

where

(5) $$K_{index}=\frac{C}{H\times \frac{R_{bit}}{N_{bit}}/\sum_{j=1}^{N_{v}}\tau_{j}}.$$

Please note that the fair-access index of a vehicle is jointly impacted by the resident time and successful transmission probability of the vehicle. It is an important index to evaluate the fairness of the proposed scheme in this paper. Specifically, to ensure fair access, each vehicle should keep the same value of $K_{index}$.

From Equation (4), we can observe that $K_{index}$ is related to the successful transmission probability $p_{s}^{i}$ and vehicle velocity $v_{i}$. To achieve fairness, we need to figure out the relationship among $K_{index}$, the velocity and minimum contention window of a vehicle. Since a vehicle can successfully transmit a packet only when no other vehicles occupy the channel, the successful transmission probability $p_{s}^{i}$ can be expressed as

(6) $$p_{s}^{i}=\tau_{i}{\left(1-\tau_{i}\right)}^{n_{i}-1}\prod_{k=1,k\neq i}^{N}{\left(1-\tau_{k}\right)}^{n_{k}}.$$

To ensure the analytical tractability, considering the condition that the number of vehicles on lane *i*, i.e., $n_{i}$, is large, ${\left(1-\tau_{i}\right)}^{n_{i}-1}$ in Equation (6) can be approximated by ${\left(1-\tau_{i}\right)}^{n_{i}}$ and Equation (6) can be simplified as

(7) $$p_{s}^{i}\approx \tau_{i}\prod_{i=1}^{N}{\left(1-\tau_{i}\right)}^{n_{i}}.$$

From Equation (7), we can find that $p_{s}^{i}$ is related to the average number of vehicles on lane *i* and the transmission probability $\tau_{i}$. Thus, we need to derive how the average number of vehicles on a lane and transmission probability $\tau_{i}$ can be expressed by the velocity and minimum content window of each vehicle in the following sub-sections.

### 4.2. Average Number of Vehicles

**Figure 2:**
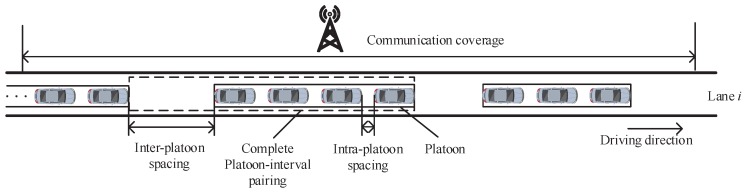
Platoon-interval pairing.

In this sub-section, we discuss the relationship among the average number of vehicles in the network on lane *i*, velocity and minimum contention window of a vehicle on lane *i*. The platoons follow one after another with an inter-platoon spacing, i.e., the interval between two consecutive platoons. As described in the system, a platoon enters the network when the headstock of the leader vehicle in the platoon enters the network. Therefore, the average distance between two consecutive platoons when the front platoon reaches the network is the sum of the average platoon length and the average inter-platoon spacing. We consider the average length of a platoon and the following interval as a platoon-interval pairing, to facilitate the analysis of the average number of vehicles in the network. The platoon-interval pairing is shown in Figure 2.

**Figure 3:**
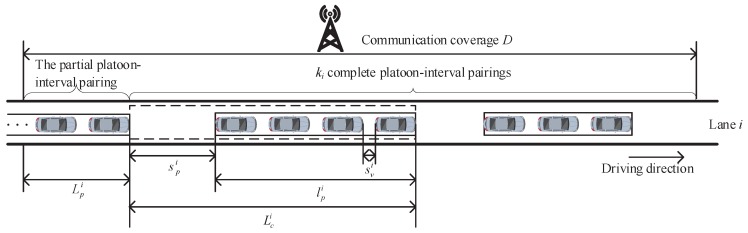
Partial platoon-interval pairing and complete platoon pairing.

Figure 3 illustrates the platoon-interval pairings on lane *i*. As shown in Figure 3, the average number of vehicles on lane *i* is the total number of the vehicles in the $k_{i}$ complete platoon-interval pairings and the partial platoon-interval pairing, i.e.,

(8) $$n_{i}=n_{c}^{i}+n_{p}^{i}.$$

We first derive the average number of vehicles in $k_{i}$ complete platoon-interval pairings. Let $m_{v}$ be the average number of vehicles in a platoon. We can obtain

(9) $$n_{c}^{i}=k_{i}m_{v}.$$

Since the number of vehicles in each platoon is independently and uniformly distributed in [a,b], the average number of vehicles in a platoon can be expressed as $m_{v}=\left(a+b\right)/2$. In Equation (9), the average number of complete platoon-interval pairings $k_{i}$ can be determined by the ratio of the infrastructure’s coverage to the average length of a platoon-interval pairing. Since $k_{i}$ is an integer, the ratio should be rounded downward to the nearest integer, i.e.,

(10) $$k_{i}=\left\lfloor \frac{D}{L_{c}^{i}}\right\rfloor .$$

Let $l_{p}^{i}$ be the average length of a platoon and $s_{p}^{i}$ be the average inter-platoon spacing. The average length of a complete platoon-interval pairing can be calculated as

(11) $$L_{c}^{i}=l_{p}^{i}+s_{p}^{i},$$

where the average length of a platoon consists of $m_{v}$ vehicle lengths and $m_{v}-1$ intra-platoon spacings. Let $s_{v}^{i}$ be the intra-platoon spacing. The average length of a platoon can be calculated as

(12) $$l_{p}^{i}=m_{v}l+\left(m_{v}-1\right)s_{v}^{i}=\left(m_{v}-1\right)\left(l+s_{v}^{i}\right)+l.$$

Substituting Equations (11) and (12) into (10), we have

(13) $$k_{i}=\left\lfloor \frac{D}{\left(m_{v}-1\right)\left(l+s_{v}^{i}\right)+l+s_{p}^{i}}\right\rfloor .$$

Substituting Equation (13) into (9), we obtain the average number of vehicles in $k_{i}$ complete platoon-interval pairings,

(14) $$n_{c}^{i}=\left\lfloor \frac{D}{\left(m_{v}-1\right)\left(l+s_{v}^{i}\right)+l+s_{p}^{i}}\right\rfloor m_{v}.$$

Next, we will derive the average number of vehicles in the partial platoon-interval pairing. The average length of the partial platoon-interval pairing is the difference between the infrastructure coverage and the average length of the $k_{i}$ complete platoon-interval pairings, i.e.,

(15) $$L_{p}^{i}=D-k_{i}\left(l_{p}^{i}+s_{p}^{i}\right).$$

As described in the system model, the transceiver is installed at the headstock of each vehicle. In this case, a vehicle is considered to enter the network once the headstock of the vehicle arrives at the communication’s coverage. Moreover, there is an intra-platoon spacing between two consecutive vehicles in a platoon. Therefore, the distance between two consecutive vehicles entering the network is the sum of a vehicle length and an intra-platoon spacing. According to Figure 3, $n_{p}^{i}$ can be determined by the ratio of the average length of the partial platoon-interval pairing to the combination of a vehicle length and an intra-platoon spacing. Since $n_{p}^{i}$ is an integer, the ratio should be rounded upward to the nearest integer. Please note that the maximum average number of vehicles in the partial platoon-interval pairing should not be larger than the average number of vehicles in a platoon. The average number of vehicles in the partial platoon-interval pairing can be calculated as

(16) $$n_{p}^{i}=\left\lceil \frac{L_{p}^{i}}{l+s_{v}^{i}}\right\rceil ,n_{p}^{i}\leq m_{v}.$$

Substituting Equations (12) and (15) into (16), we obtain the average number of vehicles in the partial platoon-interval pairing,

(17) $$n_{p}^{i}=\left\lceil \frac{D-k_{i}\left[\left(m_{v}-1\right)\left(l+s_{v}^{i}\right)+l+s_{p}^{i}\right]}{l+s_{v}^{i}}\right\rceil ,n_{p}^{i}\leq m_{v},$$

where $k_{i}$ can be calculated according to Equation (13).

In Equation (8), the average number of vehicles in the complete platoon-interval pairings and the partial platoon-interval pairing can be expressed as Equations (14) and (17), respectively. Substituting Equations (9) and (17) into (8), we have

(18) $$n_{i}=k_{i}m_{v}+\left\lceil \frac{D-k_{i}\left[\left(m_{v}-1\right)\left(l+s_{v}^{i}\right)+l+s_{p}^{i}\right]}{l+s_{v}^{i}}\right\rceil ,n_{p}^{i}\leq m_{v},$$

where $k_{i}$ can be calculated according to Equation (13).

According to Equation (18), the average number of vehicles covered by an infrastructure $n_{i}$ is related to the average inter-platoon spacing $s_{p}^{i}$ and the intra-platoon spacing $s_{v}^{i}$. Thus, we need to further analyze how to translate $s_{v}^{i}$, $s_{p}^{i}$ into $v_{i}$ and $W_{0}^{i}$.

We first derive the relationship among $s_{v}^{i}$, $v_{i}$ and $W_{0}^{i}$. Considering that the vehicles in the platoons on lane *i* are at equilibrium point [31], i.e., the velocities of all vehicles on lane *i* keep a constant $v_{i}$, the relationship between the intra-platoon spacing and the velocity is expressed as [33]

(19) $$s_{v}^{i}=\frac{s_{0}+v_{i}T_{h}}{\sqrt{1-{\left(\frac{v_{i}}{v_{0}}\right)}^{4}}}.$$

Now we will derive the relationship among $s_{p}^{i}$, $v_{i}$ and $W_{0}^{i}$. Similar with [30], we assume that the minimum inter-platoon spacing is the intra-platoon spacing. As described at the beginning of this sub-section, the average distance between two consecutive platoons when the front platoon reaches the network is the sum of the average platoon length $l_{p}^{i}$ and the average inter-platoon spacing $s_{p}^{i}$. Therefore, the time difference for the two consecutive platoons to enter the network is

(20) $$\Delta T_{i}=\frac{l_{p}^{i}+s_{p}^{i}}{v_{i}},s_{p}^{i}\leq s_{v}^{i},$$

where the average inter-platoon spacing $s_{p}^{i}$ should not be larger than the intra-platoon spacing $s_{v}^{i}$ to avoid collision.

According to the definition of the platoon arrival rate [34], we have

(21) $$\lambda_{i}=\frac{1}{\Delta T_{i}}=\frac{v_{i}}{l_{p}^{i}+s_{p}^{i}},s_{p}^{i}\leq s_{v}^{i}.$$

Substituting Equation (12) into (21), we have

(22) $$s_{p}^{i}=\frac{v_{i}}{\lambda_{i}}-\left[\left(m_{v}-1\right)\left(l+s_{v}^{i}\right)+l\right],\lambda_{i}\leq \frac{v_{i}}{m_{v}\left(l+s_{v}^{i}\right)},$$

where the platoon arrival rate can be achieved by analyzing the traffic flow recording in [35,36]. Moreover, from Equation (22), we can obtain $\lambda_{max}^{i}=\frac{v_{i}}{m_{v}\left(l+s_{v}^{i}\right)}$ when the average inter-platoon spacing reaches the minimum value, i.e., the intra-platoon spacing. In other words, when the average inter-platoon spacing is equal to the intra-platoon spacing (24), the platoon arrival rate reaches the maximum value $\lambda_{max}^{i}$.

We have obtained the relationship between $s_{v}^{i}$ and $v_{i}$ according to Equation (19) and the relationship between $s_{p}^{i}$ and $v_{i}$ according to Equation (22). Substituting Equations (19) and (22) into (18), we can find that the average number of vehicles $n_{i}$ is only related to the velocity $v_{i}$.

### 4.3. Transmission Probability

In the last sub-section, we have obtained the relationship between the average number of vehicles in the network $n_{i}$ and the velocity $v_{i}$. In this sub-section, we will further analyze how to express the transmission probability $\tau_{i}$ by the velocity $v_{i}$ and the minimum contention window $W_{0}^{i}$.

According to the reference [14], the transmission probability in a non-saturated state can be calculated as follows,

(23) $$\tau_{i}=b{\left(0,0\right)}_{e}^{i}\left(\frac{q^{2}W_{0}^{i}}{\left(1-p_{i}\right)\left(1-q\right)\left(1-{\left(1-q\right)}^{W_{0}^{i}}\right)}-\frac{q^{2}P_{idle}^{i}}{1-q}\right),$$

(24) $$\begin{array}{cc} \frac{1}{b{\left(0,0\right)}_{e}^{i}}= & \left(1-q\right)+\frac{q^{2}W_{0}^{i}\left(W_{0}^{i}+1\right)}{2[1-{\left(1-q\right)}^{W_{0}^{i}}]}+\frac{q(W_{0}^{i}+1)}{2(1-q)}\\ & \cdot [\frac{q^{2}W_{0}^{i}}{1-{\left(1-q\right)}^{W_{0}^{i}}}+\left(1-P_{idle}^{i}\right)\left(1-q\right)]\\ & -\frac{q(W_{0}^{i}+1)}{2(1-q)}\cdot qP_{idle}^{i}\left(1-p_{i}\right)+\frac{p_{i}q^{2}}{2\left(1-p_{i}\right)\left(1-q\right)}\\ & \cdot [\frac{W_{0}^{i}}{1-{\left(1-q\right)}^{W_{0}^{i}}}-\left(1-p_{i}\right)P_{idle}^{i}]\\ & \cdot [2W_{0}^{i}\frac{1-p_{i}-p_{i}{\left(2p_{i}\right)}^{m-1}}{1-2p_{i}}+1], \end{array}$$

where $b{\left(0,0\right)}_{e}^{i}$ denotes the stationary probability of the state that a vehicle on lane *i* has no packet waiting for transmission when the time counter decreases to 0; *q* denotes the probability that there is at least one packet waiting for transmission when the time counter begins to decrease; $W_{0}^{i}$ denotes the minimum contention window of a vehicle on lane *i*; $P_{idle}^{i}$ denotes the probability that the channel is detected as idle during a time slot for the vehicle on lane *i*; $p_{i}$ denotes the collision probability of the vehicle on lane *i*.

From Equations (23) and (24), we can see that the transmission probability is related to the minimum contention window size.

Since a collision occurs when multiple vehicles transmit packets at the same time, the collision probability $p_{i}$ can be expressed as

(25) $$\begin{array}{cc} p_{i} & =1-{\left(1-\tau_{i}\right)}^{n_{i}-1}\prod_{k=1,k\neq i}^{N}{\left(1-\tau_{k}\right)}^{n_{k}}. \end{array}$$

We consider the condition when the number of vehicles on lane *i* is large, and thus $p_{i}$ can be approximated to

(26) $$p_{i}\approx 1-\prod_{k=1}^{N}{\left(1-\tau_{k}\right)}^{n_{k}}.$$

Moreover, since that the channel is detected as idle when no other vehicles are transmitting in the time slot, $P_{idle}^{i}$ can be expressed as

(27) $$P_{idle}^{i}={\left(1-\tau_{i}\right)}^{n_{i}-1}\prod_{k=1,k\neq i}^{N}{\left(1-\tau_{k}\right)}^{n_{k}}=1-p_{i}.$$

Considering that the minimum competition window of a vehicle on lane *i* is much larger than 1, we have $1-{\left(1-q\right)}^{W_{i}}\approx 1$. Then $\tau_{i}$ in Equation (23) can be rewritten as

(28) $$\begin{array}{cc} \tau_{i} & =\frac{b{{\left(0,0\right)}_{e}}^{i}W_{0}^{i}}{1-q}\cdot \left\{\frac{q^{2}}{\left(1-p_{i}\right)\left[1-{\left(1-q\right)}^{W_{0}^{i}}\right]}-\frac{q^{2}\left(1-p_{i}\right)}{W_{0}^{i}}\right\}\\ & \approx \frac{b{{\left(0,0\right)}_{e}}^{i}W_{0}^{i}}{1-q}\cdot \left\{\frac{q^{2}}{\left(1-p_{i}\right)}-\frac{q^{2}\left(1-p_{i}\right)}{W_{0}^{i}}\right\}. \end{array}$$

Please note that $q^{2}\left(1-p_{i}\right)\ll 1$ and $W_{0}^{i}\gg 1$, and then $\frac{q^{2}\left(1-p_{i}\right)}{W_{0}^{i}}$ can be omitted. Thus, we can obtain that

(29) $$\tau_{i}\approx b{\left(0,0\right)}_{e}^{i}W_{0}^{i}\cdot \frac{q^{2}}{\left(1-p_{i}\right)\left(1-q\right)}.$$

To further remove $b{\left(0,0\right)}_{e}^{i}$ from the above equation, we divide both side of Equation (24) by $W_{0}^{i}$, and consider that $P_{idle}^{i}=1-p_{i}$ and $1-{\left(1-q\right)}^{W_{i}}\approx 1$. Thus, Equation (24) can be transformed into

(30) $$\begin{array}{cc} \frac{1}{b{\left(0,0\right)}_{e}^{i}W_{0}^{i}}\approx & \frac{1-q}{W_{0}^{i}}+\frac{q^{2}\left(W_{0}^{i}+1\right)}{2}+\frac{q(W_{0}^{i}+1)}{2(1-q)}\\ & \cdot \left[q^{2}+\frac{p_{i}\left(1-q\right)}{W_{0}^{i}}-\frac{q{\left(1-p_{i}\right)}^{2}}{W_{0}^{i}}\right]\\ & +\frac{p_{i}q^{2}}{2\left(1-p_{i}\right)\left(1-q\right)}\cdot \left[1-\frac{{\left(1-p_{i}\right)}^{2}}{W_{0}^{i}}\right]\\ & \cdot \left[2W_{0}^{i}\cdot \frac{1-p_{i}-p_{i}{\left(2p_{i}\right)}^{m-1}}{1-2p_{i}}+1\right]. \end{array}$$

In the case that $W_{0}^{i}\gg 1$, $\frac{1}{b{\left(0,0\right)}_{e}^{i}W_{0}^{i}}$ can be approximated as

(31) $$\begin{array}{ccc} \frac{1}{b{\left(0,0\right)}_{e}^{i}W_{0}^{i}} & \approx & \frac{q^{2}W_{0}^{i}}{2}+\frac{q^{3}W_{0}^{i}}{2(1-q)}+\frac{p_{i}q^{2}}{2\left(1-p_{i}\right)\left(1-q\right)}\\ & & +\frac{p_{i}q^{2}W_{0}^{i}}{\left(1-p_{i}\right)\left(1-q\right)}\cdot \frac{1-p_{i}-p_{i}{\left(2p_{i}\right)}^{m-1}}{1-2p_{i}}\\ & = & W_{0}^{i}\cdot \left[\frac{q^{2}}{2}+\frac{q^{3}}{2(1-q)}\right]\\ & & +W_{0}^{i}\cdot \frac{p_{i}q^{2}}{\left(1-p_{i}\right)\left(1-q\right)}\cdot \frac{1-p_{i}-p_{i}{\left(2p_{i}\right)}^{m-1}}{1-2p_{i}}\\ & & +\frac{p_{i}q^{2}}{2\left(1-p_{i}\right)\left(1-q\right)}. \end{array}$$

Substituting Equation (31) into (29), we have

(32) $$\tau_{i}\approx \frac{1}{W_{0}^{i}\times \left\{\frac{1-p_{i}}{2}+\frac{p_{i}\left[1-p_{i}-p_{i}{\left(2p_{i}\right)}^{m-1}\right]}{1-2p_{i}}\right\}+\frac{p_{i}}{2}}.$$

Because $\tau_{i}\ll 1$, $1/\tau_{i}\gg 1$, and thus $p_{i}/2$ is a negligible value. As a result, the transmission probability $\tau_{i}$ is eventually approximated by

(33) $$\tau_{i}\approx \frac{1}{W_{0}^{i}\times f\left(p_{i}\right)},$$

where

(34) $$f\left(p_{i}\right)=\frac{1-p_{i}}{2}+\frac{p_{i}\left[1-p_{i}-p_{i}{\left(2p_{i}\right)}^{m-1}\right]}{1-2p_{i}}.$$

From Equations (33) and (34), it can be seen that when the minimum contention window is large, the transmission probability is very small and is independent of *q*. Substituting Equation (33) into (26), we can find $p_{i}$ is related to $W_{0}^{i}$ and $n_{i}$. Since $n_{i}$ is related to $v_{i}$ according to the results obtained in Section 4.2, $p_{i}$ is related to $W_{0}^{i}$ and $v_{i}$. Moreover, since $\tau_{i}$ is related to $W_{0}^{i}$ and $p_{i}$ according to Equation (26) and $p_{i}$ is related to $W_{0}^{i}$ and $v_{i}$. Therefore, $\tau_{i}$ is related to $W_{0}^{i}$ and $v_{i}$. Finally, we can derive the relationship among the transmission probability, the vehicle velocity, and the minimum contention window.

### 4.4. Velocity-Adaptive V2I Fair-Access Scheme

In Section 4.2, we have obtained the relationship between the average number of vehicles in the network $n_{i}$ and the velocity $v_{i}$. In Section 4.3, we have derived the relationship among the transmission probability, velocity, and minimum contention window. Thus, $K_{index}$ can be expressed as a function of $v_{i}$ and $W_{0}^{i}$.

Substituting Equation (26) into (7), we have

(35) $$\begin{array}{cc} p_{s}^{i} & \approx \tau_{i}\prod_{i=1}^{N}{\left(1-\tau_{i}\right)}^{n_{i}}\\ & =\tau_{i}\left(1-p_{i}\right). \end{array}$$

Substituting Equations (33) and (35) into (4), we can derive the relationship between $K_{index}$ and the minimum contention window when $T_{i}$ is given as $\frac{D}{v_{i}}$, i.e.,

(36) $$\begin{array}{cc} K_{index} & =p_{s}^{i}T_{i}\\ & =\frac{1-p_{i}}{W_{0}^{i}f\left(p_{i}\right)}\cdot \frac{D}{v_{i}}\;\\ & =\frac{D}{W_{0}^{i}v_{i}X\left(p_{i}\right)}, \end{array}$$

where

(37) $$X\left(p_{i}\right)=\frac{f(p_{i})}{1-p_{i}}=\frac{1}{2}+\frac{p_{i}\left[1-p_{i}-p_{i}{\left(2p_{i}\right)}^{m-1}\right]}{\left(1-p_{i}\right)\left(1-2p_{i}\right)}.$$

Please note that $X(p_{i})$ is only related to the collision probability $p_{i}$. According to the results obtained in Section 4.3, $p_{i}$ is related to $W_{0}^{i}$ and $v_{i}$. Comprehensively, $X(p_{i})$ depends on $v_{i}$ and $W_{0}^{i}$.

Given that *D* is a constant, to keep $K_{index}$ as a constant, $W_{0}^{i}v_{i}X\left(p_{i}\right)$ should also be a constant. Thus, we mark $W_{0}^{i}v_{i}X\left(p_{i}\right)$ as a constant $K_{c}$, i.e.,

(38) $$K_{c}=W_{0}^{i}v_{i}X\left(p_{i}\right)=\frac{D}{K_{index}}.$$

Averaging the two sides of Equation (38) and expanding to all networks, $K_{c}$ can be predefined as follows

(39) $$K_{c}=\bar{W}\times \bar{v}\times \bar{X(p)},$$

where $\bar{W}$ represents the average minimum competition window of the system and $\bar{v}$ represents the average velocity of the system which can be obtained based on the distribution of the velocity. Since $X(p_{i})$ depends on $v_{i}$ and $W_{0}^{i}$, we can obtain $\bar{X(p)}$ through $\bar{W}$ and $\bar{v}$.

Combining Equations (38) and (39), the minimum contention window size $W_{0}^{i}$ can be adjusted according to the velocity $v_{i}$ to keep the same value of $K_{index}$, i.e., achieve fairness, for each vehicle.

### 4.5. Normalized Throughput

In this sub-section, we analyze the normalized throughput of the proposed scheme.

According to [14], the normalized throughput of the system is defined as

(40) $$\begin{array}{cc} H & =\frac{E[payload\;information\;successfully\;transmitted\;in\;a\;slot\;time]}{E[length\;of\;a\;slot\;time]}\\ & =\frac{P_{t}\left(1\right)E\left[P\right]}{P_{t}\left(0\right)\sigma +P_{t}\left(1\right)T_{s}+\left[1-P_{t}\left(0\right)-P_{t}\left(1\right)\right]T_{c}}, \end{array}$$

where E[P] represents the average load size of a data packet; $P_{t}\left(1\right)$ represents the probability of a successful transmission in a slot time, that is, only one vehicle occupies the channel; $P_{t}\left(0\right)$ represents the probability that there is no vehicle transmitting in a slot time; $1-P_{t}\left(0\right)-P_{t}\left(1\right)$ represents the probability of a collision in a slot time; $T_{s}$ indicates the average duration of a successful transmission in a slot time; $T_{c}$ indicates the average duration of a collision in a slot time; σ indicates the duration of an empty slot time.

According to the analysis in Section 4 and Equation (40), it can be observed that the calculation of normalized throughput is very complicated. Since that the transmission probability is very small, we can approximate the binomial distribution to the Poisson distribution [34]. Thus, we can use the Poisson distribution to describe the above process and approximate the throughput. Please note that when there are more nodes in the network, the approximation method is more accurate. The approximate method is shown as follows:

Define $P_{t}\left(y\right)$ as the probability that there are *y* nodes sending packets in the same time slot.

(41) $$P_{t}\left(y\right)=e^{-\lambda_{t}}\frac{\lambda_{t}^{y}}{y!},$$

where $\lambda_{t}$ is the Poisson distribution arrival rate of packets, that is, the expected number of data packets transmitted in a time slot. Therefore, we have

(42) $$\lambda_{t}=\sum_{i=1}^{N}n_{i}\tau_{i},$$

where $n_{i}$ can be obtained from Section 4.2, and $\tau_{i}$ can be obtained from Section 4.3.

Since that $P_{t}\left(0\right)$ represents the probability that there is no node transmitting in a slot time, $P_{t}\left(0\right)$ can be written as

(43) $$P_{t}\left(0\right)=e^{-\lambda_{t}}.$$

Since that a successful transmission means that there is only one node transmitting packets, $P_{t}\left(1\right)$ can be written as

(44) $$P_{t}\left(1\right)=\lambda_{t}e^{-\lambda_{t}}.$$

Since that $1-P_{t}\left(0\right)-P_{t}\left(1\right)$ represents the probability of a collision in a slot time, $1-P_{t}\left(0\right)-P_{t}\left(1\right)$ can be written as

(45) $$1-P_{t}\left(0\right)-P_{t}\left(1\right)=1-e^{-\lambda_{t}}-\lambda_{t}e^{-\lambda_{t}}.$$

Substituting the above three equations into Equation (40), the normalized throughput can be simplified as

(46) $$H=\frac{\lambda_{t}e^{-\lambda_{t}}E\left[P\right]}{e^{-\lambda_{t}}\sigma +\left(1-e^{-\lambda_{t}}-\lambda_{t}e^{-\lambda_{t}}\right)T_{c}+\lambda_{t}e^{-\lambda_{t}}T_{s}}.$$

## 5. Simulation Results

In this section, we consider the highway scenario described in Section 3 with two lanes and four lanes, respectively. We verify the relationship between the performance of the fair-access scheme and the average velocity of different lanes under the different platoon arrival rates $\lambda_{max}$ and $0.8\lambda_{max}$ through simulation experiments. As described in Section 3, the maximum value $\lambda_{max}$ is the platoon arrival rate when the average inter-platoon spacing is equal to the intra-platoon spacing (24). We also consider that the speed limit of American highway ranges from 20 m/s to 30 m/s, which can be obtained from [37]. Therefore, we set the average velocity of the system as 25 m/s. In addition, the average minimum contention window of the system is 64. In the two-lane scenario, vehicles drive on the two lanes in the same direction and the velocity difference between the two lanes is always 4 m/s, e.g., when the average velocity of two lanes is 22.5 m/s, the velocity of vehicles driving on lane 1 is 20.5 m/s, and the velocity of vehicles driving on lane 2 is 24.5 m/s. Similarly, in the four-lane scenario, vehicles drive on lane 1 and lane 2 in the same direction and vehicles drive on lane 3 and lane 4 in the opposite direction. The velocities of vehicles driving on lane 1 and lane 4 are the same, and the velocities of vehicles driving on lane 2 and lane 3 are the same. The velocity difference between lane 1 and lane 2 is always 4 m/s. The simulation tool is MATLAB-R2014b. The parameters used in the simulation are listed in Table 2.

**Figure 4:**
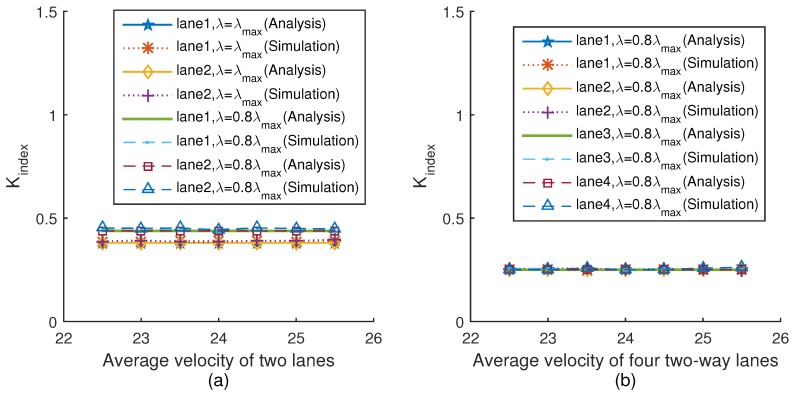
Fairness index versus velocity.

Figure 4a,b show the relationship between fairness index $K_{index}$ and the average velocity of two lanes and four lanes under two different platoon arrival rates, respectively. The simulation results are very close to the theoretical values. It can be seen from Figure 4a that when the platoon arrival rate is $\lambda_{max}$, the fairness index $K_{index}$ of two lanes is the same. Moreover, with the increment of average velocity, $K_{index}$ is still unchanged, which means that the total amount of data successfully transmitted by each vehicle on the two lanes is equal. When the platoon arrival rate is equal to $0.8\lambda_{max}$, the fairness index $K_{index}$ keeps unchanged with the velocity increasing. Thus, the fairness can be guaranteed by the proposed scheme. This is attributed to the fact that when the velocity of a vehicle changes, the vehicle dynamically adjusts the minimum contention window to keep the fairness index unchanged, which is consistent with Equation (36). The trend of Figure 4b is the same as Figure 4a.

**Figure 5:**
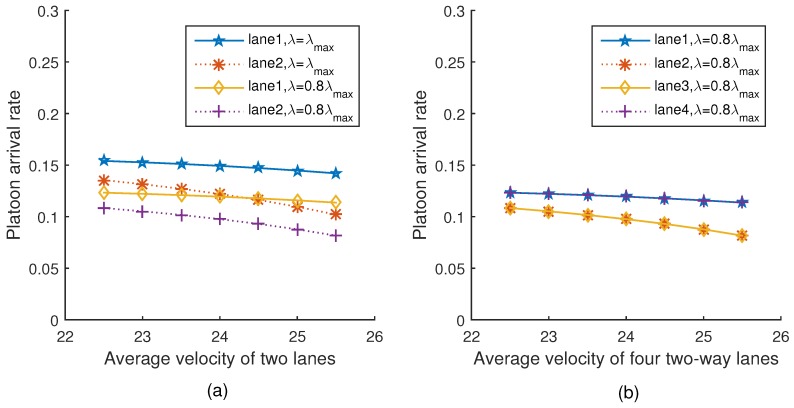
Platoon arrival rate versus velocity.

Figure 5a,b show the trend of platoon arrival rates when the average velocity of two lanes and four lanes varies under two different platoon arrival rates, respectively. It can be seen from Figure 5a that with the average velocity increasing, both the two different platoon arrival rates of vehicles decrease. This is because the intra-platoon spacing and the inter-platoon spacing increase with the increment of vehicle velocity in the network, which would further cause a decrement of the platoon arrival rate. This is consistent with Equation (21). The trend of Figure 5b is the same as Figure 5a.

**Figure 6:**
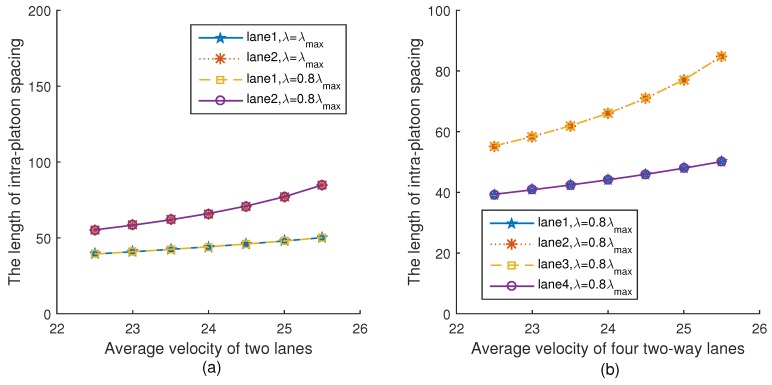
The intra-platoon spacing versus velocity.

Figure 6a,b show the relationship between the intra-platoon spacing and the average velocity of two lanes and four lanes under two different platoon arrival rates, respectively. It can be seen from Figure 6a that for the same lane, the intra-platoon spacing is always the same under different platoon arrival rates. This is because according to Equation (19), the intra-platoon spacing is only related to velocity, but not to the platoon arrival rate. In addition, as the velocity increases, the intra-platoon spacings of the vehicles on both lanes increase, which is consistent with Equation (19). The trend of Figure 6b is the same as Figure 6a.

**Figure 7:**
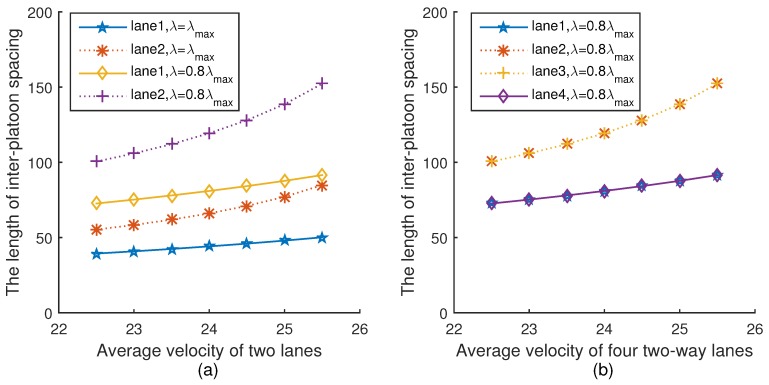
The inter-platoon spacing versus velocity.

Figure 7a,b show the relationship between the inter-platoon spacing and the average velocity of two lanes and four lanes under two different platoon arrival rates, respectively. Please note that when $\lambda =\lambda_{max}$, the length of inter-platoon spacing is equal to the intra-platoon spacing in Equation (22), and thus the inter-platoon spacing is the minimum safe distance. It can be seen from Figure 7a that the length of inter-platoon spacing under $\lambda_{max}$ is less than that under $0.8\lambda_{max}$. This is because when the velocity and the intra-platoon spacing are given in Equation (19), with the platoon arrival rate decreasing, the inter-platoon spacing increases in Equation (22). In addition, with the average velocity increasing, the length of inter-platoon spacing increases, which is consistent with Equation (22). The trend of Figure 7b is the same as Figure 7a.

**Figure 8:**
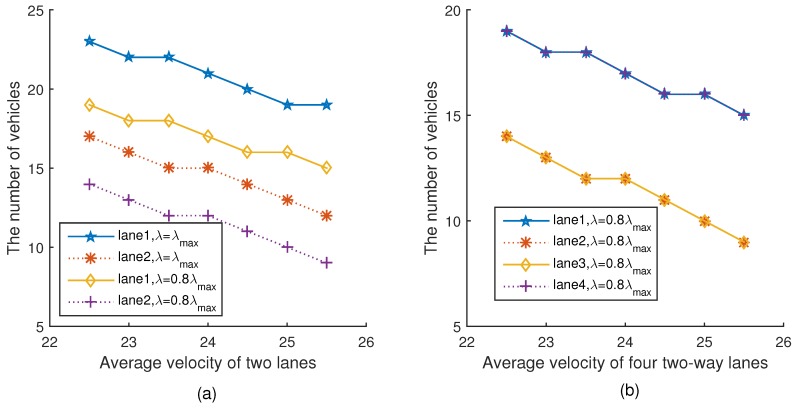
The number of vehicles versus velocity.

Figure 8a,b show the relationship between the number of vehicles in the network and the average velocity of two lanes and four lanes under two different platoon arrival rates, respectively. Please note that for the same lane, the number of vehicles under $0.8\lambda_{max}$ is less than that under $\lambda_{max}$. It can be seen from Figure 8a that with the average velocity increasing, the numbers of vehicles in the network under the two different platoon arrival rates also decrease. This is because with the increment of velocity, the inter-platoon spacing is extended as shown in Figure 7, thus reducing the number of vehicles in the network, which is consistent with Equations (21) and (22). The trend of Figure 8b is the same as Figure 8a.

**Figure 9:**
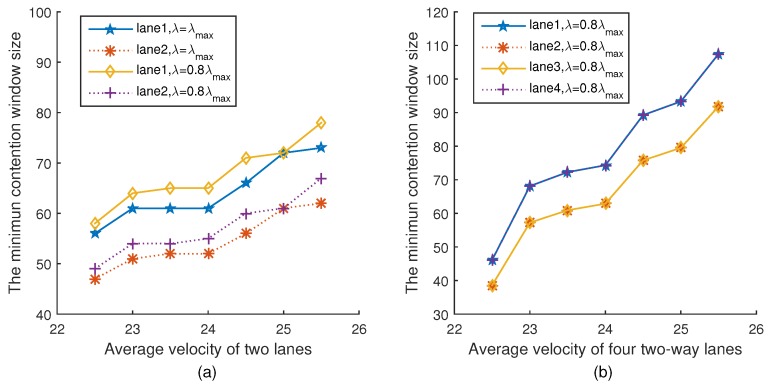
Minimum contention window versus velocity.

Figure 9a,b show the relationship between the minimum contention window size and the average velocity of two lanes and four lanes under two different platoon arrival rates, respectively. It can be seen from Figure 9a that with the average velocity increasing, the minimum contention window size increases. This is because when the velocity of a vehicle changes, the vehicle dynamically adjusts the minimum contention window to keep the fairness index unchanged, which is consistent with Equation (36). The trend of Figure 9b is the same as Figure 9a.

**Figure 10:**
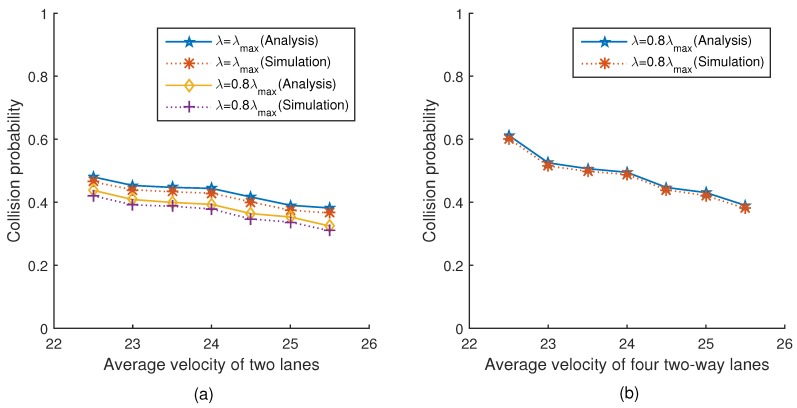
Collision probability versus velocity.

Figure 10a,b show the relationship between the collision probability and the average velocity of two lanes and four lanes under two different platoon arrival rates, respectively. The simulation results are very close to the theoretical values. Please note that the curve in the figure is not smooth, because the number of vehicles in the network decreases un-smoothly, which is obvious in Equation (26). It can be seen from Figure 10a that with the average velocity increasing, the collision probability gradually decreases. This is because with the average velocity increasing, the number of vehicles in the network constantly decreases, thus reducing the collision probability. Similarly, the collision probability under $\lambda_{max}$ is lower than that under $0.8\lambda_{max}$, because the number of vehicles in the network under $\lambda_{max}$ is more than that under $0.8\lambda_{max}$. The trend of Figure 10b is the same as Figure 10a.

**Figure 11:**
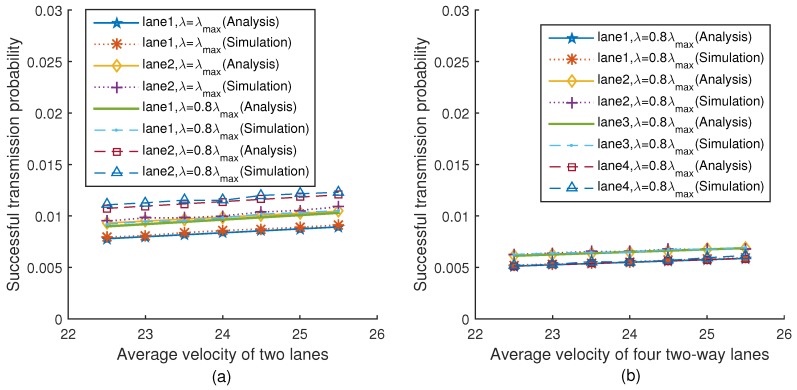
Successful transmission probability versus velocity (q=0.3).

Figure 11a,b show the relationship between the successful transmission probability and the average velocity of two lanes and four lanes under two different platoon arrival rates respectively when q=0.3. The simulation results are very close to the theoretical values. It can be seen from Figure 11a that with the average velocity increasing, the successful transmission probability increases correspondingly. This is because with the average velocity increasing, the number of vehicles in the network keeps decreasing, thus reducing the collision probability, which impacts the successful transmission probability. In addition, from Figure 11a, we can find that for each lane, the successful transmission probability under $\lambda_{max}$ is lower than that under $0.8\lambda_{max}$. This is because the number of vehicles in the network under $\lambda_{max}$ is more than that under $0.8\lambda_{max}$, and thus the successful transmission probability is lower, which is consistent with Equation (35). The trend of Figure 11b is the same as Figure 11a.

**Figure 12:**
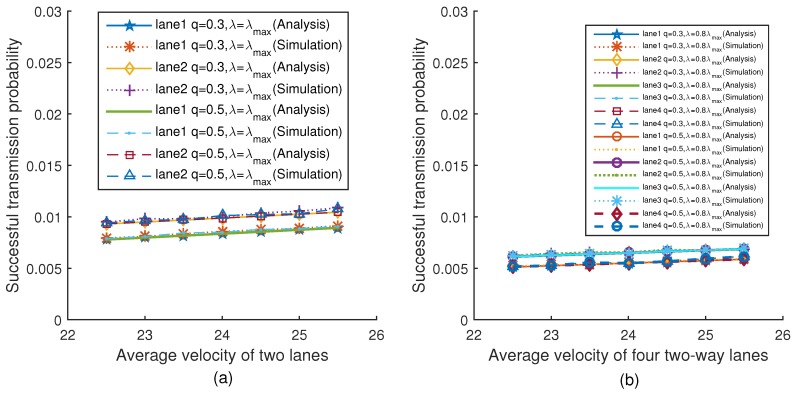
Successful transmission probability versus velocity.

Figure 12a,b show the relationship between the successful transmission probability and the average velocity of two lanes and four lanes under two different platoon arrival rates respectively (when q=0.3 and q=0.5). The simulation results are very close to the theoretical values. It can be seen from Figure 12a that although the values of *q* are different, the successful transmission probability of vehicles on the same lane is the same when the velocity is given. This is because the transmission probability is not related to *q* in Equation (32). The successful transmission probability is only related to the transmission probability and collision probability in Equation (35). Moreover, the collision probability is only related to the transmission probability and the average number of vehicles in Equation (26). Therefore, the successful transmission probability is independent of *q*. The trend of Figure 12b is the same as Figure 12a.

**Figure 13:**
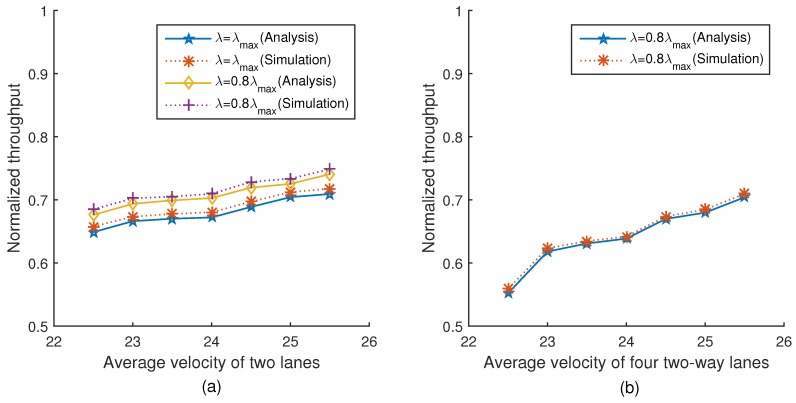
Normalized throughput versus velocity.

Figure 13a,b show the relationship between the normalized throughput and the average velocity of two lanes and four lanes under two different platoon arrival rates, respectively. The simulation results are very close to the theoretical values. It can be seen from Figure 13a that with the average velocity increasing, the normalized throughput increases correspondingly. This is because with the average velocity increasing, the number of vehicles in the network constantly decreases, leading to a reduced collision probability which improves the normalized throughput. We can also find that the normalized throughput under $\lambda_{max}$ is lower than that under $0.8\lambda_{max}$. This is because the number of vehicles in the network under $\lambda_{max}$ is more than that under $0.8\lambda_{max}$, and thus the collision probability is larger, and the normalized throughput is lower, which is consistent with Equation (46). The trend of Figure 13b is the same as Figure 13a.

## 6. Conclusions

In this paper, we proposed the velocity-adaptive V2I fair-access scheme based on IEEE 802.11 DCF for platooning vehicles. The proposed scheme ensures that all vehicles successfully transmit data with equal probability by dynamically adjusting the minimum contention window according to the velocity. We proved that if vehicles have the same fair-access index, these vehicles access the roadside infrastructure fairly. We also analyzed the normalized throughput of the proposed scheme. The effectiveness of our scheme has been validated by simulation. In future work, we will continue to study the practicability and scalability of the proposed scheme and try to study the relevant fair-access scheme for the communications between vehicles in platoons.

## Figures and Tables

**Table 1:** Parameters used in the analytical model.

$b{\left(0,0\right)}_{e}^{i}$	The stationary probability of the state that a vehicle on lane *i* has no packet waiting for transmission when the time counter decreases to 0
*D*	The infrastructure coverage
E[P]	The average load size of the data packet
*H*	The normalized throughput of the network
$K_{index}$	The fairness index
$k_{i}$	The number of complete platoon-interval pairings on lane *i*
$L_{c}^{i}$	The average length of a complete platoon-interval pairing on lane *i*
$L_{p}^{i}$	The average length of the partial platoon-interval pairing on lane *i*
*l*	The average length of a vehicle
$l_{p}^{i}$	The average length of a platoon on lane *i*
*m*	The maximum back-off stage
$m_{v}$	The average number of vehicles in a platoon
*N*	The number of lanes in the network
$N_{bit}$	The average number of bits in a packet
$N_{v}$	The total number of vehicles in the network
$n_{i}$	The number of vehicles on lane *i*
$n_{c}^{i}$	The number of vehicles in $k_{i}$ complete platoon-interval pairings on lane *i*
$n_{p}^{i}$	The number of vehicles in the partial platoon-interval pairing on lane *i*
$P_{t}\left(0\right)$	The probability that there is no node transmitting in a slot time
$P_{t}\left(1\right)$	The probability of a successful transmission in a slot time
$P_{idle}^{i}$	The probability that the channel is detected idle during a time slot of a vehicle on lane *i*
$p_{i}$	The collision probability of a vehicle on lane *i*
$p_{s}^{i}$	The successful transmission probability of a vehicle on lane *i*
*q*	The probability that there is at least one packet waiting for transmission when the time counter begins to decrease
$R_{s}^{i}$	The successful transmission rate of a vehicle on lane *i*
$s_{p}^{i}$	The average inter-platoon spacing on lane *i*
$s_{v}^{i}$	The intra-platoon spacing on lane *i*
$s_{0}$	The minimum intra-platoon spacing
$T_{h}$	The time headway
$T_{i}$	The residence time of a vehicle within the communication coverage of the infrastructure on the *i*th lane
$v_{i}$	The velocity of a vehicle driving on lane *i*
$v_{0}$	The maximum velocity of a vehicle in the network
$W_{0}^{i}$	The minimum contention window of a vehicle on lane *i*
$\Delta T_{i}$	The time difference between two consecutive platoons on lane *i* to enter the network
$\lambda_{i}$	The platoon arrival rate on lane *i*
$\lambda_{t}$	The Poisson distribution arrival rate of packets
$\lambda_{max}^{i}$	The maximum platoon arrival rate on lane *i*
$\tau_{i}$	The transmission probability of a vehicle on lane *i*

**Table 2:** Simulation parameter settings.

ACK (μs)	240
D (m)	1000
DIFS (μs)	128
E[P] (bits)	8184
*l* (m)	5
*m*	3
$m_{v}$	3
*N*	2/4
SIFS (μs)	28
Slot time (μs)	50
$s_{0}$ (m)	2
$T_{c}$ (bits)	8713
$T_{s}$ (bits)	8972
$T_{h}$ (s)	1.6
$v_{0}$ (m/s)	30
$\bar{v}$ (m/s)	25
$\bar{W}$	64
